# Exploration of demographic prevalence of mild cognitive impairment using Montreal cognitive assessment: A cross-sectional pilot study in the Cape Coast Metropolis, Ghana.

**DOI:** 10.1016/j.ibneur.2024.11.008

**Published:** 2024-11-20

**Authors:** David Larbi Simpong, George Nkrumah Osei, Richeal Odarko Mills, Christopher Amaleyele Anyebem, Benjamin Kofi Aikins, Charlotte Gyanwaa Melfah, Bridget Amoanimaa Osei, Ansumana Bockarie

**Affiliations:** a Department of Medical Laboratory Science, School of Allied Health Sciences, University of Cape Coast, Cape Coast, Ghana; b Department of Biomedical Science, School of Allied Health Sciences, University of Cape Coast, Cape Coast, Ghana; c Department of Internal medicine and Therapeutics, School of Medical Sciences, University of Cape Coast, Cape Coast, Ghana

**Keywords:** Mild Cognitive Impairment, Dementia, Montreal Cognitive Assessment, Prevalence, Demographics

## Abstract

**Background:**

The Global prevalence of dementia is projected to rise, particularly in low and middle-income countries like Ghana. Mild cognitive impairment (MCI), an intermediate phase between normal cognitive aging and dementia, is characterized by an objective and subjective decline in cognitive abilities. Individuals with MCI have a greater likelihood of progression to dementia.

**Purpose:**

There is a paucity of studies focused on assessing the prevalence, risk factors and characteristics of mild cognitive impairment within the Ghanaian population. This study assessed the prevalence of mild cognitive impairment and explored its relationship with various sociodemographic factors.

**Methods:**

A prospective cross-sectional analytical study within Cape Coast, Ghana, evaluating the cognition of 100 participants using the Montreal Cognitive Assessment (MoCA) tool. The prevalence of MCI was determined using simple descriptive measures. The two-way ANOVA was used to determine risk factors for developing MCI. The Pearson correlation coefficient was used to determine the relationship between educational level and MoCA score.

**Results:**

A majority (65.4 %) of participants within the age group 40–49 years had mild cognitive impairment. 42.86 % of male and 40.54 % of female participants had MCI (MoCA score < 26). There was a significant correlation (r= 0.608, p= 0.0001) between the educational level of participants and the MoCA score. Participants classified as having MCI based on their MoCA score, performed significantly poorer in visuospatial, attention, language, abstraction and delayed recall domains compared to those with normal cognition.

**Conclusion:**

The MoCA tool is a useful for detecting MCI, particularly among Ghanaians with at least 7 years of formal education. The prevalence of MCI among individuals aged 40–49 years in the Cape Coast Metropolis represents an important health burden.

## Introduction

Mild cognitive impairment (MCI), an intermediate phase between normal cognitive aging and dementia, is characterized by an objective and subjective decline in cognitive abilities relative to the pre-existing levels of functioning of an individual (Morris, 2005, Bai et al., 2022, Jia et al., 2021, Anderson, 2019, Manly et al., 2008, Matthews et al., 2008, Tsolaki et al., 2017, Roberts and Knopman, 2013, Lara et al., 2016, Lopez-Anton et al., 2015, Farias et al., 2009, Eshkoor et al., 2015, Nasreddine et al., 2005a). Projections indicate that the global prevalence of dementia will rise steadily in the coming decades, potentially doubling approximately every 20 years from an estimated 46.8 million cases worldwide in 2015 to over 131.5 million by 2050 (Anderson, 2019, Prince et al., 2015). Most of this substantial growth is expected to take place in low and middle-income nations (Anderson, 2019, Prince et al., 2015). The prevalence of MCI varies widely, from as low as 3 % to as high as 42 % varying across country of study origin, with lower prevalence in studies that included younger participants (Anderson, 2019, Manly et al., 2008, Petersen et al., 2018, Ward et al., 2012).

Sociodemographic factors such as increase in age, gender, lack and low level of formal education have been identified as significant determinants of mild cognitive impairment risk (Bai et al., 2022, Tsolaki et al., 2017, Tianyi et al., 2019, Ravaglia et al., 2008, Tang, 2020, Sachdev et al., 2015). Research has also shown that individuals meeting the criteria for MCI are at an approximately 12 % risk of progressing to dementia compared to only 1–2 % risk among cognitively normal individuals of the same age annually (Tsolaki et al., 2017, Petersen, 2004, Petersen, 2003). A meta-analysis of 41 cohort studies also found a cumulative 39.2 % proportion of MCI cases progressing to dementia (Bai et al., 2022, Mitchell and Shiri-Feshki, 2009). While some individuals with MCI may later demonstrate normalized cognitive function, their likelihood of recurrent MCI or progression to dementia remains greater than for those who never exhibited signs of mild cognitive impairment (Roberts and Knopman, 2013, Onwuekwe, 2012).

Of the many instruments available for the assessment of cognition, the Montreal Cognitive Assessment (MoCA) is probably the most widely and best validated tool. It has been validated in several African countries (Masika et al., 2021, Saleh et al., 2019, Daniel et al., 2022). The MoCA is a brief cognitive assessment measuring multiple domains through a single page of items. Cognitive areas evaluated include executive function and visuospatial skills, naming abilities, memory, attention, language, abstraction, delayed recall, and orientation (Dautzenberg et al., 2020). It is suggested that age and level of education may affect the utility of the MoCA in some instances, the no clear pattern identified for the effect of education. Cultural differences similarly affect the performance of the tool (Sperling et al., 2013).

Given the paucity of effective treatments for dementia and evidence that irreversible brain changes or damage occurs years before overt cognitive symptoms and diagnosis, it is crucial to explore factors that contribute to MCI incidence before its progression to dementia (Jia et al., 2021, Nyame et al., 2019). The situation in Ghana is no different. As Nyame et al. noted in 2019, more research is needed to highlight and identify potential strategies for preventing or delaying the development of dementia among Ghanaians (Nasreddine et al., 2005b). A review of the existing literature revealed the absence of studies that focused on assessing the prevalence, risk factors and characteristics of mild cognitive impairment among the Ghanaian population. This study assessed the prevalence of mild cognitive impairment and explored its relationship with various sociodemographic factors. The findings of this study will provide empirical data on mild cognitive impairment prevalence and also provide clinical insight to guide the development of public health initiatives targeting early identification and treatment interventions to delay or prevent its progression to dementia.

## Methods

### Study design, site, duration and sample size

A prospective cross-sectional analytical study was conducted in Amamoma, one of the communities neighboring the University of Cape Coast between 2nd to 27th May 2022. Amamoma is a community (1^0^ 17^’’^42–18 W 5^0^ 6’35–56’’N) located within the Cape Coast North metropolis with a population size of approximately 2000. The sample size was calculated based on an estimated 50 % prevalence of MCI, with a 10 % margin of error and a confidence level of 95 % and using the formular, [Z^2^p (1-p)]/C^2^. An estimated size of 92 was obtained and this was rounded up to 100 to compensate for participant non-response.

Participants were selected from among locals attending the community market. This was to ensure that potential participants were reached during working / day time hours to facilitate face-to-face interviews. Shop identification numbers were listed from which 100 participants were to be selected. There was a total of 260 shops, from which 100 participants were to be selected. Using systematic random sampling every third shop was approached to consent to participate in the study. This process was repeated until the estimated 100 participants was achieved.

### Eligibility criteria

Individuals aged 30 years and above with no known chronic condition or cognitive complaints were eligible for the study. Individuals with acquired brain injury, substance abuse, recent treatment for psychiatric or neurologic diseases, and use of medication that can alter cognitive functioning were excluded. Non-consenting individuals were also excluded.

### Ethical Consideration

Ethical approval was obtained from the Cape Coast Teaching Hospital Institutional Review Board (ethical clearance ID: CCTHERC/EC/2021/020). Our research protocol complied with the provisions of the Declaration of Helsinki, 1995 (as revised in 2013). Verbal consent was obtained from the participants before recruitment, after they were given a detailed explanation of the study. Participants had the right to withdraw their consent to the study at any time. Confidentiality was also observed throughout the entire study. Authors did not take any information that could identify participants during or after data collection thereby ensuring the anonymity of participants.

### Collection of demographics of participants

Demographic data including age (stratified into 30–39 years, 40–49 years, 50–59years and ≥60 years), sex (male and female) and level of formal education (no education, primary, Junior High School, Senior High School, and Tertiary) were obtained using an interviewer-administered face-to-face structured questionnaire.

### Assessment of cognitive function using the Montreal Cognitive Assessment (MoCA)

The cognition of participants was evaluated using the Montreal Cognitive Assessment (MoCA) tool version 8.3. Prior to test administration, researchers completed training on the administration and evaluation of the MoCA. Where necessary, translation of the questions was provided into local languages for those with limited English language proficiency and education. Based on this assessment tool, a participant who obtained a score of less than 26 was considered to have mild cognitive impairment, and those who scored 26 and above were considered to have normal cognition (Smith and Yeow, 2016).

### Data analysis

The prevalence of MCI was determined using simple descriptive measures such as frequencies and percentages. The two-way ANOVA was used to determine whether age or sex variables were risk factors to developing MCI. The Pearson correlation coefficient was used to determine the relationship between a person’s educational level and MoCA score. A p-value less than 0.05 was considered statistically significant.

## Results

**Fig. 1:**
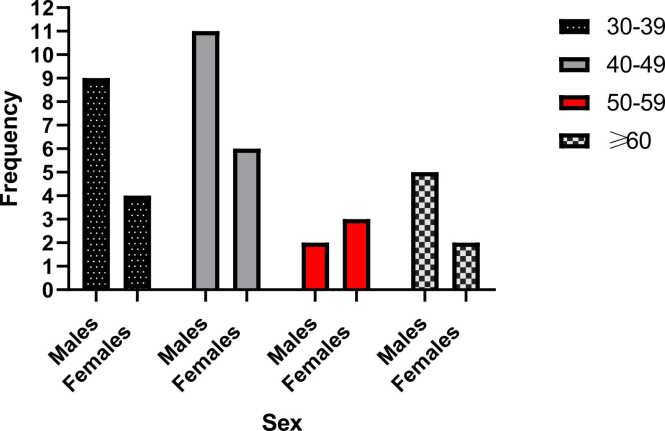
Distribution of mild cognitive impairment between sex and age group.

The study involved 100 study participants with majority being males (63 %). The mean age was 42.7±11.96 with minimum and maximum ages of 30 and 76 respectively (Table 1). The majority of the participants within age group 30–39 years [41(83.67 %)] had at least 12 years of formal education (Table 1). Using MoCA cognitive assessment tool to evaluate the cognition, 17 (65.38 %) participants within the age group of 40–49 years had mild cognitive impairment (MoCA score <26) as shown in Table 1. On evaluating cognition among sex and age groups, 42.86 % (27 out of 63) of male and 40.54 % (15 out of 37) of female participants had mild cognitive impairment (MoCA score < 26) (Fig. 1). However, a higher prevalence in of MCI was found among females [20.0 % (3/15)] within the age group of 50–59 than males [7.4 % (2/27)] (Fig. 1).

**Table 1:** Demographic and Cognitive Characteristics of Study Participants.

Age group (years)	Males	Females	<12 yrs edu	≥12 yrs edu	MoCA score < 26	MoCA score ≥ 26
Mean age, n±SD - 42.7±11.96						
30 – 39	34	16	9	41	13	37
40 – 49	15	11	14	12	17	9
50 – 59	5	5	7	3	5	5
≥ 60	9	5	11	3	7	7

SD: Standard deviation, MCI: Mild Cognitive Impairment, edu: education, yrs: years

**Fig. 2:**
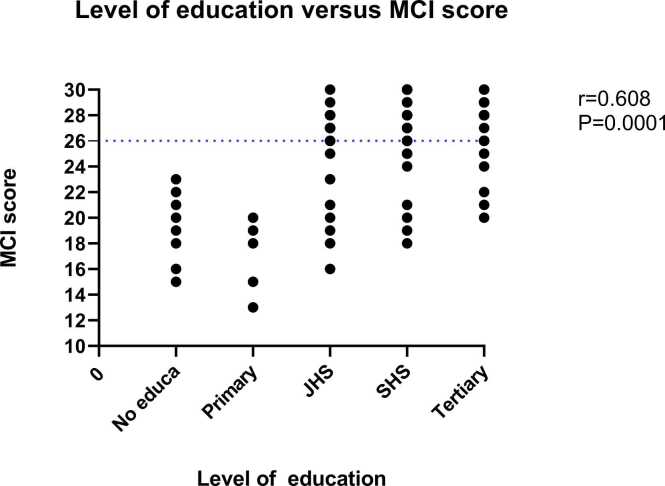
Correlation between the educational level of participants and cognitive assessment using the MoCA tool. No educa - no formal education; JHS – Junior High School; SHS – Senior High School.

There was a significant correlation (r= 0.608, p= 0.0001) between the educational level of participants and the MoCA cognitive assessment score (Fig. 2). All participants with no formal education as well as those with only primary level of education had MoCA score of < 26, suggestive of mild cognitive impairment among these categories of persons (Fig. 2). For respondents whose highest education was Junior High School (JHS) and Senior High School (SHS), 12(48.0 %) and 9(26.5 %) had mild cognitive impairment respectively (MoCA score <26) (Table 2).

**Table 2:** Distribution of MoCA Scores Based on Educational Attainment among Study Participants.

Level of education	Total count	MoCA score < 26 n(%)	MoCA score ≥ 26 n(%)
*No Education*	11	11(100.0)	-
*Primary*	5	5(100.0)	-
*JHS*	25	12(48.0)	14(52.0)
*SHS*	34	9(26.5)	24(73.5)
*Tertiary*	25	5(20.0)	20(80.0)

Of the eight variables evaluated by the MoCA cognitive assessment tool, language (1.87/3) and delayed recall (3.46/5) showed the lowest average performance, while orientation showed the highest (5.84/6) (Table 3). However, participants who were classified as having MCI based on a MoCA score, performed significantly poorer in visuospatial, attention, language, abstraction and delayed recall domains compared to those with normal cognition (Table 3).

**Table 3:** Performance of Participants based on MoCA Assessment Domains.

		Total		MCI (MoCA score <26)	No MCI (MoCA score ≥ 26)
Areas of Assessment	**Maximum**		**Mean± SD**	**Mean± SD**	**Mean± SD**
*Visuospatial*	5.00	3.54±1.41		2.31±1.24	4.43±0.65
*Naming*	3.00	2.74±0.56		2.60±0.66	2.85±0.45
*Attention*	6.00	5.12±1.19		4.26±1.31	5.74±0.55
*Language*	3.00	1.87±1.12		0.98±0.98	2.52±0.68
*Abstraction*	2.00	1.52±0.69		1.21±0.84	1.74±0.44
*Delayed Recall*	5.00	3.46±1.39		2.55±1.40	4.12±0.94
*Orientation*	6.00	5.84±0.49		5.71±0.67	5.93±0.26

SD: Standard deviation, MCI: Mild Cognitive Impairment.

## Discussion

This study assessed the prevalence of mild cognitive impairment in an urban settlement in Ghana and its relationship with demographic factors such as age, sex, and education level.

The prevalence of MCI was found to be 42 % in our study. This prevalence is higher than that observed in similar studies by Bai et al. (2022) [15.56 %], Lara et al. (2016)[9.6 %], Jia et al. (2021)[36.2 %], and Ravaglia et al. (2008)[7.7 %] globally, in Spain, China and Italy respectively (Bai et al., 2022, Jia et al., 2021, Lara et al., 2016, Ravaglia et al., 2008). However, our finding is lower than observed by Smith & Yeow in 2016 [56 %] (Sosa et al., 2012). Variations in the prevalence of MCI across studies could be due to differences sociodemographic features of the populations studied, disease prevalence, definitions and diagnostic criteria used (Song et al., 2023, Fisk et al., 2003, Au et al., 2017).

Our study revealed a relatively even distribution of mild cognitive impairment between the sexes, 42.86 % of males (27 out of 63), and 40.54 % of females (15 out of 37). Inferring that both males and females have comparable risk for mild cognitive impairment. This finding aligns with the findings reported by Au et al. (2017) and Sachdev et al. (2015), which found no statistically significant sex differences in the prevalence of MCI (Sachdev et al., 2015, Langa et al., 2017). This however differs from the results of Lara et al. (2016) and Jia et al. (2021), which observed lower MCI prevalence among males (Jia et al., 2021, Lara et al., 2016). These variations could be due to differences in sample characteristics such as size, research methodology employed, and diagnostic approach utilized across the studies.

Subgroup analysis by age revealed that the majority (65.38 %) of participants between 40 and 49 years exhibited mild cognitive impairment. This draws attention to middle age as a critical period for emerging MCI risks. This is important, considering individuals at this stage are typically engaged in careers upon which both themselves, their families and their communities rely for years to come. However, unlike prior research by Jia et al. (2021), Bai et al. (2022), and Ravaglia et al. (2008) which found increased MCI prevalence in those aged above 75, 80 and 84 years respectively (Bai et al., 2022, Jia et al., 2021, Ravaglia et al., 2008), our findings draw attention to the economically prodcutive, middle age group (between 40 and 49 years) as another key life stage for increased MCI susceptibility. The lower life expectancy in Ghana compared to countries like Italy might account for this earlier presentation of MCI. Recognizing that nearly two-thirds of our 40–49-year-old participants displayed MCI symptoms signifies the importance of screening this population, to better support long-term individual and social outcomes.

In consonance with studies by Bai et al. (2022) and Ravaglia et al. (2008) which found an increased prevalence of MCI among participants with ≤6 (19.75 %) and ≤ 3 years of education (14.4 %), our study also observed higher prevalence of MCI in individuals with no formal education or only primary education. This elevated risk may be due to the fact that higher education attainment enables the development of stronger cognitive reserves through continued mental stimulation in formal schooling, thereby shielding against age-related cognitive declines (Bai et al., 2022; $author1$ et al.,). Notably, almost half of junior high school graduates in our study still met diagnostic criteria for MCI, signifying that risk persists even among those with that level of education. This however also raises the question as to whether the MoCA tool is suitable for evaluation of MCI among person with no formal education or less those with less than 7 years of formal education. Acceptance of the findings would highlight individuals with no education or less than 7 years of formal education as priority targets for intervention efforts. A Chilean study similarly found education and age but not sex to significantly impact on MoCA scores [^39^].

Participants classified as having MCI in this study exhibited significantly lower performance on tests of visuospatial, attention, language, abstraction, and delayed recall abilities compared to those displaying normal cognition. Specifically, language, visuospatial, and delayed recall domains revealed the lowest mean scores amongst the MCI group. This finding lends useful insight, suggesting deficits in these areas may serve as early indicators of cognitive changes in individuals with MCI. In contrast, orientation and naming skills were observed to be relatively preserved even in individuals meeting criteria for MCI. This suggests that orientation functions and naming capabilities tend to be among the last faculties impacted as cognition progresses from normal to the MCI stage. This aligns with the fact that; orientation is impacted later in relation to other cognitive capacities.

This study had a few limitations. First, the study design was cross-sectional, and thus cannot infer causation of MCI. Additionally, our study was not designed to generate clinical diagnoses for dementia and hence some individuals with dementia may have been inadvertently incorporated into the mild cognitive impairment case group. Although this study was community based, the small size and a single site makes generalization of the findings to other contexts difficult.

## Conclusion

This study shows that the MoCA tool is useful for detecting MCI particularly, among those with at least 7 years of formal education in Ghana. The prevalence of MCI among individuals in the Cape Coast Metropolis especially, those aged 40–49 years represent an important societal burden for this category of people. These subjects, who are at the prime of their careers and professional lives, are at greater risk of dementia in the future. The current findings also contribute to an increased understanding of the prevalence and characteristics of MCI in a representative sample of the Ghanaian population. It forms the basis of the justification for a larger, multi-centre study, with the aim of standardising the MoCA for the assessment of MCI in the Ghanaian population. This would allow for follow up, longitudinal research, to examine the progression from MCI to dementia and identify modifiable risk and protective factors.

## Funding Statement

This study received no external funding.

## ICMJE Statement

All authors agree to be accountable for all aspects of the work in ensuring that questions related to the accuracy or integrity of any part of the work are appropriately investigated and resolved

## Ethical Statement

Ethical approval was obtained from the Cape Coast Teaching Hospital Institutional Review Board (ethical clearance ID: CCTHERC/EC/2021/020). Our research protocol complied with the provisions of the Declaration of Helsinki, 1995 (as revised in 2013). Verbal informed consent was obtained from the participants before recruitment and documented.

## Ethical statement

Ethical approval was obtained from the Cape Coast Teaching Hospital Institutional Review Board (ethical clearance ID: CCTHERC/EC/2021/020). Our research protocol complied with the provisions of the Declaration of Helsinki, 1995 (as revised in 2013). Verbal consent was also obtained from the participants before recruitment, after they were given a detailed explanation of the study. Participants had the right to withdraw their consent to the study at any time. Confidentiality was also observed throughout the entire study. Authors did not take any information that could identify participants during and after data collection thereby ensuring the anonymity of participants.

## CRediT authorship contribution statement

**David Larbi Simpong:** Writing – review & editing, Writing – original draft, Validation, Supervision, Resources, Project administration, Methodology, Investigation, Formal analysis, Data curation, Conceptualization. **Benjamin Kofi Aikins:** Writing – review & editing, Validation, Resources, Project administration, Methodology, Investigation, Formal analysis, Data curation. **Christopher Amaleyele Anyebem:** Writing – review & editing, Software, Resources, Methodology, Investigation, Formal analysis, Data curation. **Richael Odarkor Mills:** Writing – original draft, Validation, Software, Project administration, Methodology, Investigation, Formal analysis. **George Nkrumah Osei:** Writing – original draft, Validation, Methodology, Investigation, Formal analysis, Data curation. **Ansumana Bockarie:** Writing – review & editing, Validation, Supervision, Software, Methodology, Investigation, Formal analysis, Data curation, Conceptualization. **Bridget Amoanimaah Osei:** Writing – review & editing, Validation, Resources, Methodology, Investigation, Formal analysis, Data curation. **Charlotte Gyanwaa Melfah:** Writing – review & editing, Validation, Resources, Project administration, Methodology, Investigation, Data curation.

## Declaration of Competing Interest

The authors declare that they have no known competing financial interests or personal relationships that could have appeared to influence the work reported in this paper.

## Data Availability

All data generated or analyzed during this study are available upon request from the corresponding author.
